# The eSMAF: a software for the assessment and follow-up of functional autonomy in geriatrics

**DOI:** 10.1186/1471-2318-7-2

**Published:** 2007-02-13

**Authors:** Patrick Boissy, Simon Brière, Michel Tousignant, Eric Rousseau

**Affiliations:** 1 Research Centre on Aging, Sherbrooke Geriatric University Institute, Sherbrooke, Quebec, Canada; 2 Université de Sherbrooke, Faculty of Physical and Sports Education, Kinesiology Dept., Sherbrooke, Quebec, Canada; 3 Université de Sherbrooke, Faculty of Medicine and Health Sciences, Rehabilitation Dept., Sherbrooke, Quebec, Canada; 4 Université de Sherbrooke, Faculty of Medicine and Health Sciences, Dept. of Physiology and Biophysics, Sherbrooke, Quebec, Canada; 5 Etienne Le Bel Research Center, CHUS, Biomedical Telematics Laboratory, Respiratory Health Network of the FRSQ, Sherbrooke, Quebec, Canada

## Abstract

**Background:**

Functional status or disability forms the core of most assessment instruments used to identify mix and level of resources and services needed by older adults who possess common characteristics. The Functional Autonomy Measurement System (SMAF) is a 29-item scale measuring functional ability in five different areas. It has been recommended for use for home care, for allocation of chronic beds, for developing care plans in institutional settings and for epidemiological and evaluative studies. The SMAF can also be used with a case-mix classification system (Iso-SMAF) to allocate resources based on patients' functional autonomy characteristics. The objective of this project was to develop a software version of the SMAF to facilitate the evaluation of the functional status of older adults in health services research and to optimize the clinical decision-making process.

**Results:**

The eSMAF was developed over an 24-month period using a modified waterfall software engineering process. Requirements and functional specifications were determined using focus groups of stakeholders. Different versions of the software were iteratively field-tested in clinical and research environments and software adaptations made accordingly. User documentation and online help were created to assist the deployment of the software. The software is available in French or English versions under a 30-day unregistered demonstration license or a free restricted registered academic license. It can be used locally on a Windows-based PC or over a network to input SMAF data into a database, search and aggregate client data according to clinical and/or administrative criteria, and generate summary or detailed reports of selected data sets for print or export to another database.

**Conclusion:**

In the last year, the software has been successfully deployed in the clinical workflow of different institutions in research and clinical applications. The software performed relatively well in terms of stability and performance. Barriers to implementation included antiquated computer hardware, low computer literacy and access to IT support. Key factors for the deployment of the software included standardization of the workflow, user training and support.

## Background

Demographic changes are leading to increased demand for services and resources in the continuum of geriatric care. With the emergence of Integrated Service Delivery Systems to improve continuity of care between settings, clinicians and managers need reliable assessment instruments capable of identifying elders' needs, promoting the provision of the appropriate resources, and providing standardized data that can match elders and providers across the continuum [1]. Functional status or disability forms the core of most assessment instruments used to identify mix and level of resources and services needed by older adults who possess common characteristics.

In the province of Quebec, Canada, a battery of assessment tools was implemented in 2001 across the entire continuum of geriatric care. Included in this package, the Functional Autonomy Measurement System (SMAF) [2] is used in clinical settings for the assessment and follow-up of elderly disabled patients [3,4]. The SMAF [see Additional File 1] is a 29-item scale based on the World Health Organization classification of disabilities [5]. It measures functional ability in five areas: activities of daily living (ADL) (7 items), mobility (6 items), communication (3 items), mental functions (5 items), and instrumental activities of daily living (IADL) (8 items). Each item is scored on a 4-point scale from 0 (independent) to 3 (dependent) for a maximum score of 87. For every item that has a rating of 1 or higher (i.e. not independent), the human resources (help or supervision) required to overcome the disability in this specific area and the stability of these resources is evaluated. The SMAF must be administered by a trained health professional, who scores the individual after obtaining the information by questioning the subject and proxies or by observing the subject. The theoretical framework of functional autonomy used for the development of the SMAF is presented elsewhere [6]. A newer theoretical framework taking into account the social aspects of disability and documenting the impact of the social and physical environment on a person's functioning has been proposed since [7,8]. The impact of social roles on functional autonomy is currently not included in the SMAF evaluation. A subscale assessing social functioning has been developed [9] and is currently being evaluated for addition to the SMAF.

The SMAF has been recommended for use for home care, for allocation of chronic beds, for developing care plans in institutional settings and for epidemiological and evaluative studies [10]. The reliability, validity and responsiveness of the SMAF has been established extensively [11-14]. Recently, a new case-mix classification was developed to better allocate resources based on the functional autonomy characteristics of patients as indicated by the SMAF [15,16]. The classification [see Additional File 2] matches client results on the 5 dimensions of the SMAF scale to 14 mutually exclusive profiles (Iso-SMAF) using Euclidean distance measure. SMAF and Iso-SMAF profiles are associated with a specific amount of nursing and support services and also with the cost of services according to the type of facility [17].

Health service researchers have routinely used these instruments as outcome measures in program evaluation and economic evaluation of geriatric health services [10,18-21]. Traditionally, this is done using paper-based clinical pathways where information from paper forms is converted into a format suitable for computerized quantitative data analysis by manual data entry. Both SMAF and Iso-SMAF are currently incorporated in dedicated clinical information systems. However, current clinical information systems that incorporate SMAF and Iso-SMAF data are not easily available to clinicians or researchers outside large public institutions (i.e. private seniors' homes, assisted living facilities). These systems offer limited interoperability and user-based data management functions with no way to search and view aggregated SMAF and Iso-SMAF data for a given client or group of clients. In order to address these specific needs, a stand-alone software was developed to increase the accessibility of these tools to the research and academic community. This paper describes the development of the software, its functions and how it is currently being used. The software is available in French or English versions under a 30-day unregistered demonstration license or a free restricted registered academic license through the Sherbrooke Health Expertise Center. Download instructions are listed in the availability and requirements section at the end of the paper.

## Methods

### Software engineering and implementation

**Figure 1:**
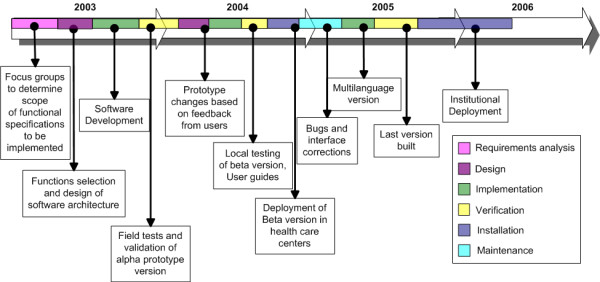
Timeline in the last 3 years for the development, implementation and deployment of the eSMAF. Specific steps within the modified waterfall model are color-coded as indicated in the legend on the right.

The development of the eSMAF followed a modified waterfall model with feedback loops from users. The modified waterfall model is a software development model in which development is seen as flowing steadily downwards (like a waterfall) through the phases of requirements analysis and functional specifications, design and implementation of software architecture, software testing and validation, and software deployment and maintenance [22]. Each phase has a well-defined starting and ending point with a closed feedback loop with identifiable deliverables to the next phase. The eSMAF project started in 2003 and culminated in the last year with the commercial deployment of the software in several organizations and institutions. The timeline for the development phases of the eSMAF is illustrated in Figure 1. The details of the development, implementation and deployment of the software are described below.

#### Requirements analysis and functional specifications

At the outset of this project, the requirements for the proposed software were to deliver in a timely manner (15 months) and with limited financial resources (about US$30 K) a low maintenance software application to: 1) facilitate SMAF data collection at the point of care, and 2) provide a way for clinicians, managers and researchers to easily access, search and view aggregated SMAF and Iso-SMAF data for a given client or group of clients. The priority was to develop a French version of the software first and then consider multi-language versions (the SMAF evaluation has been translated into 7 languages). The software was to be used under different research and clinical environments with limited IT support by single or multiple users (less than 25 users at the same time). Functional specifications for the software were established through focus groups (n = 3, 20 individuals total) of stakeholders (MD, nurses, PT, OT, department head, hospital managers, health service researchers) and prioritized by expert consensus with the development team according to the initial requirements list, analyses of feasibility (level of difficulty to implement) and development cost of each feature, and overall impact on the software architecture.

#### Design and implementation of software architecture

**Figure 2:**
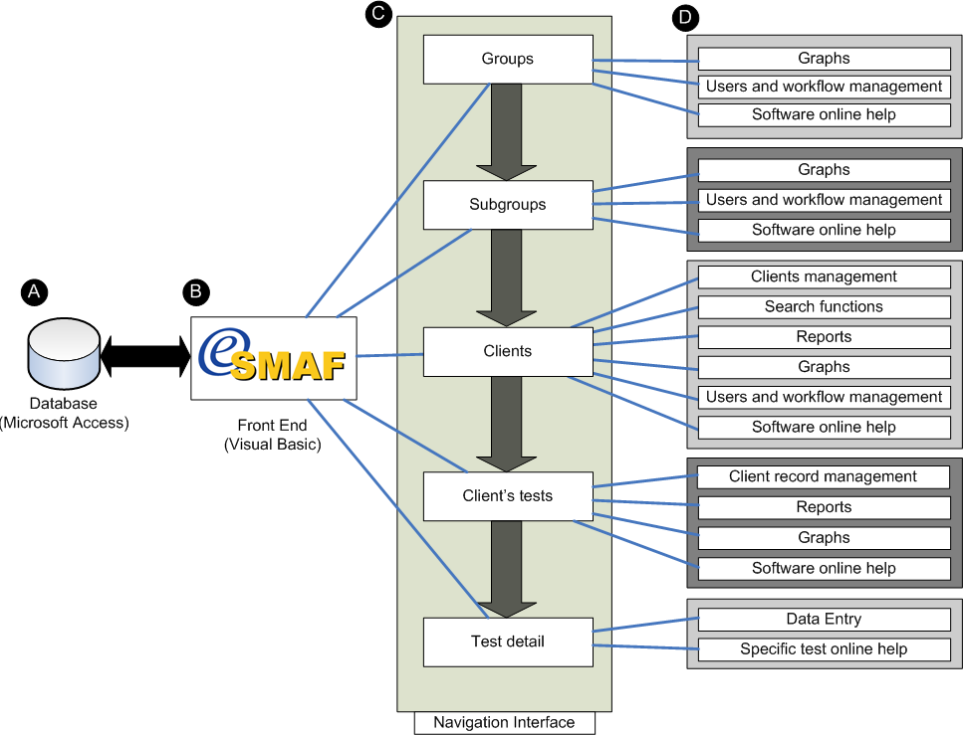
Software architecture of the eSMAF. a) Microsoft Access database is used as a back end to the software; b) eSMAF shell programmed in Visual Basic is used as a front end to display and enter data in the database; c) Hierarchical levels within the eSMAF shell; and d) Functions available in the software depending on the hierarchical levels.

After taking into consideration the complexities of the information flow of the SMAF data (from data entry to consultation to report and data extraction by clinical personnel, managers and researchers) and its variations depending on the context of use (homecare, hospital care or research), very early in the project we abandoned the idea of embedding the eSMAF in existing proprietary clinical information systems currently in use. In order to transmit information from this stand-alone system back to the patient's electronic medical record or other databases, we chose static merging of individual or aggregated group data from the eSMAF database as CVS files using an export function. An overview of the software architecture for the eSMAF is summarized in Figure 2. In keeping with the initial requirements list and projected use of the software, we chose as the basis for the software architecture 1) Microsoft Access as a back end (database) to store information, and 2) a Visual Basic client to act as a front end (software interface) for easy data insertion, retrieval and reporting. The format of the database was selected according to projected deployment constraints (limited availability of on-site and remote technological support and cost issues) and ease of installation (no server required).

The software architecture is based on five overlapping hierarchical levels (group, subgroup, client, client test, test detail). Each level provides a different view of the database (ex: global aggregated results at the group level to detailed individual information at the test detail level) from which specific functions such as user and workflow management, client record management, online help, graphs, reports, data extraction or printing can be executed. The core of the software architecture is the "Clients" level where a unique subject identifier is created, tests or data entry masks are linked to this unique identifier (Client Test level) and data entry functions (Test edition) are applied. The unique identifier at the "Clients" level can be associated with "Groups" or "Subgroups" to aggregate clients' records in user-defined categories, thus facilitating navigation and viewing of records in the database. For example, a group could be used to represent a health care center with the subgroups representing the different units in this center. In this way, the user can view individual patient records for a subgroup of clients in an institution. The group and subgroup can also be used to limit access to records to a specific category of user.

#### Software testing and validation

Testing and validation of the software was done iteratively throughout the software development process. Feedback from users regarding the usability of the software was elicited at the different stages of development and testing of the eSMAF software. Usability is defined as the extent to which a product can be used by specified users to achieve specific goals effectively, efficiently, and with satisfaction in a specified context of use [23]. The first version was completed at the end of 2003 and tested in-house over a 2-month period at the Sherbrooke Geriatric University Institute with a selected group of beta testers (n = 12 nurses) as they evaluated 600 residents in the long-term care units of two facilities. Beta testers were asked to keep a journal logging usability issues and any bugs. They were then debriefed as a group or individually by the project manager to identify software instability (i.e. bugs) and obtain suggestions for improvements or additional features.

A list of changes to be implemented was formulated and the software went through another design phase at the beginning of 2004. Existing features were optimized and new ones added. At the end of 2004, a new version was built and distributed to a larger group of beta testers. Software robustness was tested through a large-scale deployment in 19 separate institutions (approximately 200 users in total) as part of a 2-year research project on health services [24]. During this testing phase, usability issue and bugs identified by the users in the 19 institutions taking part in the trial (see discussion section) were transmitted to the development team by the field project coordinator and changes were made periodically until a stable version was obtained. In 2005, the need for a multi-language (French and English) version of the eSMAF arose. This version, which was to become the last, was developed and tested in a small group of testers. As part of the software testing and validation, the need for user guides and training material (tutorials) was identified. A user guide with screen shots was written and incorporated as part of the online help function in the software or as a stand-alone document. The user guide was translated for the multi-language (French and English) version of the eSMAF.

#### Deployment and maintenance

In January 2006, the software was released for institutional users. Licensing and training on the software is supported by the Sherbrooke Health Expertise Center. User support is offered on a contractual basis through a partnership agreement with a private company. As of July 2006, approximately 35 site licenses for a total of 250 workstations have been distributed to institutions in Canada and France. Institutional licensing efforts are still ongoing.

## Results

### Software functions of eSMAF

The principal functions of the eSMAF software are summarized below and include: (1) Navigation interface and records management utility; (2) Users and workflow management; (3) Client record module and data entry; (4) Report function and care planning; (5) Search functions; and (6) Data extraction function and online help.

#### Navigation interface and records management utility

**Figure 3:**
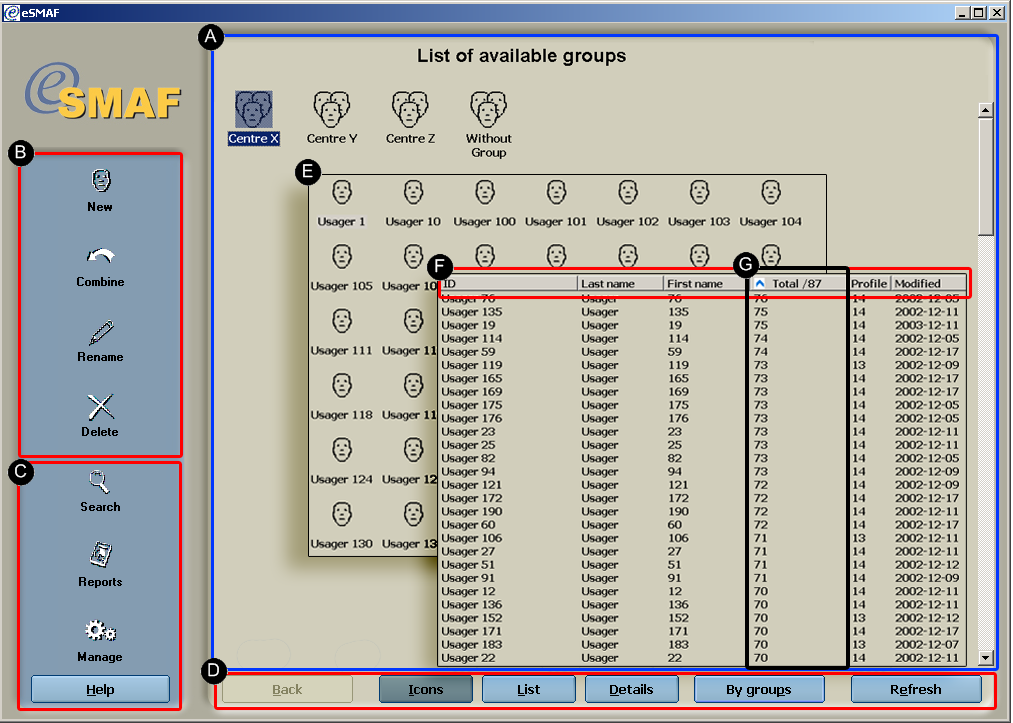
Main interface organization of eSMAF shell. a) Main area; b) Toolbar area for records management; c) Toolbar area for software functions; d) Navigation toolbar area where individual buttons are used to go back, switch display mode and refresh the display; e) Icons record view; f) Detailed record view; and g) Sorting function in detailed record view.

A standard interface is used throughout the hierarchical levels of the software. The layout of the interface is shown in Figure 3. There are three main work areas: the main area, the toolbar area and the navigation toolbar area. The main area is used to display or edit information. The toolbar area provides access to the software functions, depending on the hierarchical level the user is on. At the group level, functions include data management functions, report and search functions, and the software management control panel. The interface at the group and client level consists of buttons and list views which can be used to display patients or tests in different modes: icons, list and detailed view. The detailed view includes a sorting option that allows users to display records according to specific criteria (ex: date of creation or Iso-SMAF profile). The navigation toolbar allows users to navigate across hierarchical levels of the software.

#### Users and workflow management

**Figure 4:**
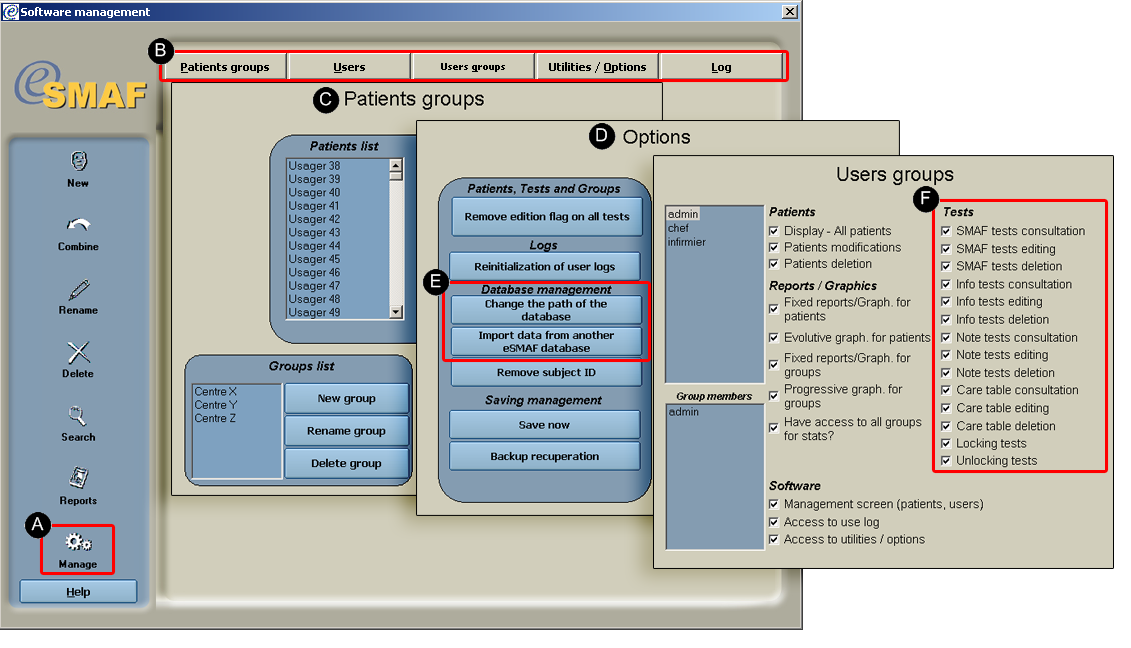
Users and workflow management. a) Access to users and workflow management; b) Control panel of the software divided into 5 sections; c) Workflow management functions enabling users to classify patient records into user-defined groups; d) Software options and preferences; and f) Management of user groups and subgroups access and editing privileges within the database.

Workflow management options (administrator and user privileges, records management, etc.) are set through a control panel accessible from the group level interface. Figure 4 shows the interface of this control panel and the principal workflow management options. Access privileges for users are defined according to user groups created by the administrator users. Each user is associated with one user group. The user group determines what kind of access the user in question has (logging into the database, accessing groups or subgroups in the client records, viewing and/or editing records or portions of records). A "Utilities/Options" panel accessible only to users with administrative privileges is used to configure options for the software (ex: path to the location of the database). Traceability features (ex: what was changed in a given record and when) can be viewed through a software log tab as all user actions are logged in the database.

#### Client record module and data entry

**Figure 5:**
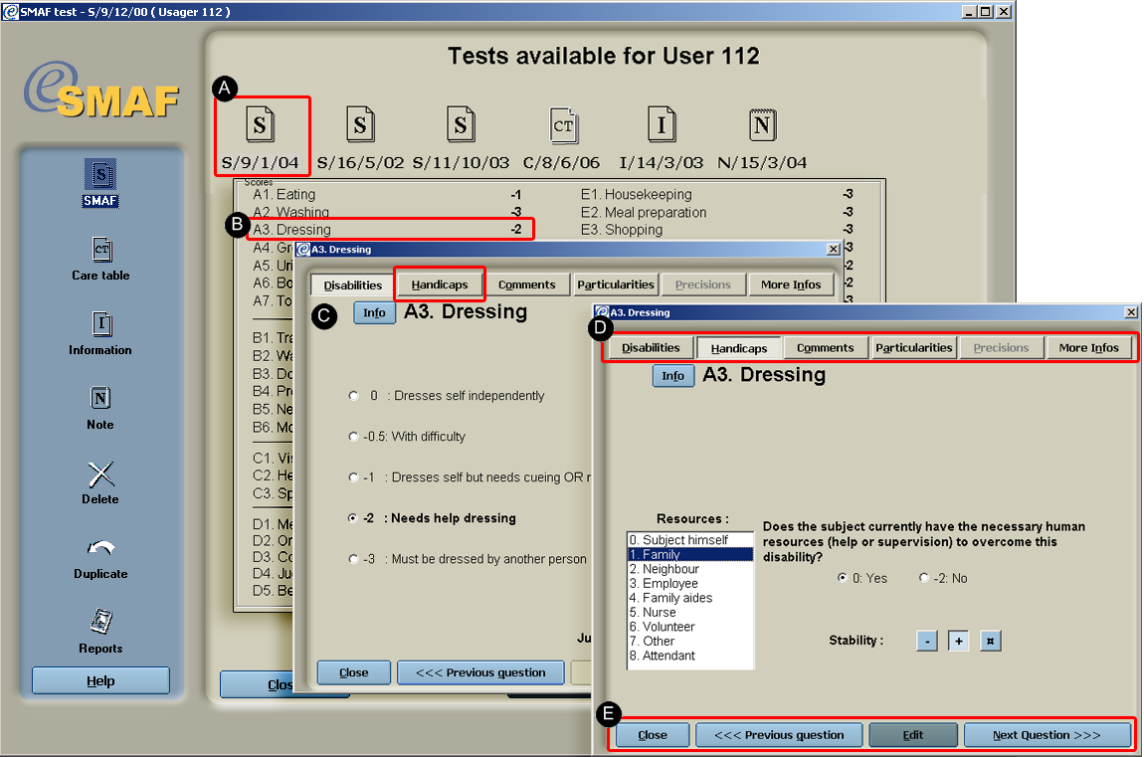
Data entry mask for SMAF evaluation. a) Upon choosing a SMAF test, the user is presented with a summary of the test; b) By activating a specific item, a data entry window appears for the selected item; c) The user can fill out the data from the test; d) More details and relevant information for this particular question can be entered using the tabs; and e) Navigation between questions and sections.

The client record module consists of a data repository of evaluations completed for a given client, allowing users with administrative privileges to generate new evaluation masks. Each evaluation can then be filed and edited. The main evaluation used in the software is the SMAF test, but it is also possible to fill out an information record containing nominal information, a clinical note and a care table (see Report function and care planning below). The SMAF test interface is shown in Figure 5. A test can be locked and unlocked, depending on the access the user has. Data are entered by accessing questions using the overall questionnaire view or using the navigation functions in the question view. Complementary information for a given item can be entered through tab navigation in the question view.

#### Report function and care planning

**Figure 6:**
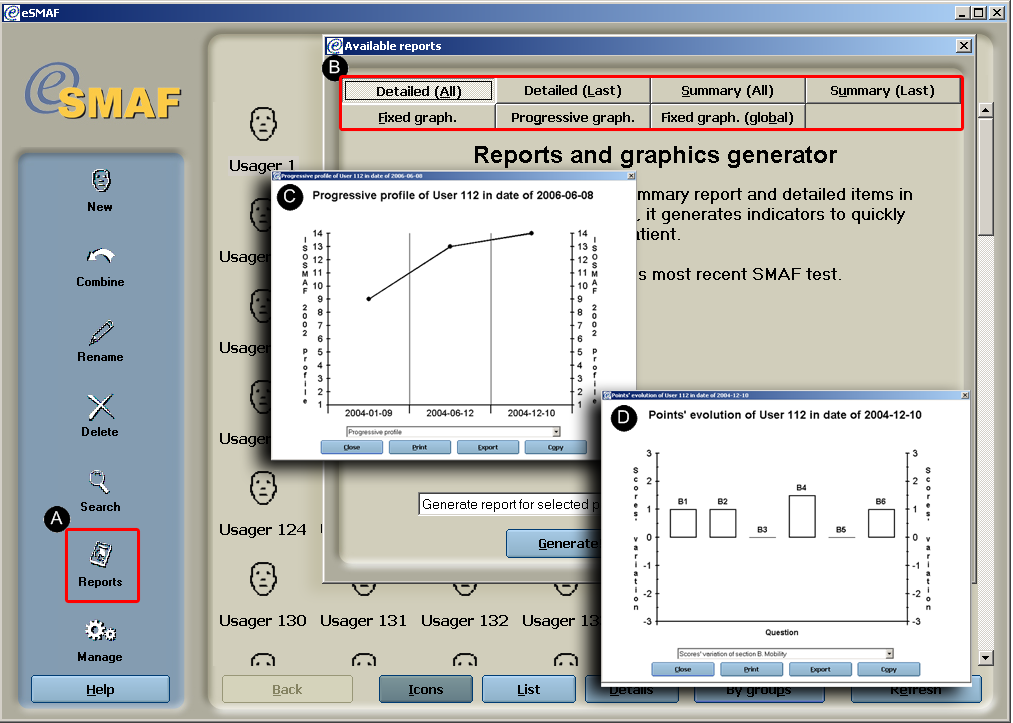
Report function. a) Buttons activating report functions; b) Data from the SMAF for a given individual or several individuals are tabulated and illustrated according to 7 types of summaries; c) and d) Evolution of a given individual's or several individuals' clinical scores can be viewed in different formats.

In order to continue support for paper-based clinical pathways, a report module was incorporated to allow users to generate paper summary reports or detailed reports on any given client or group of clients. Reports can take several forms and include: individualized summary, distribution graphs on specific indices (ex: Iso-SMAF profiles) of selected groups of patients, line graphs charting the evolution of a specific category of data for an individual across multiple evaluations, and bar charts showing specific changes between the two last evaluations of a given individual. Report functions can be applied at the client level or aggregate clients level by manually selecting them in the navigation interface and records management utilities or through selected outputs from the search engine. The principal report function outputs are illustrated in Figure 6.

**Figure 7:**
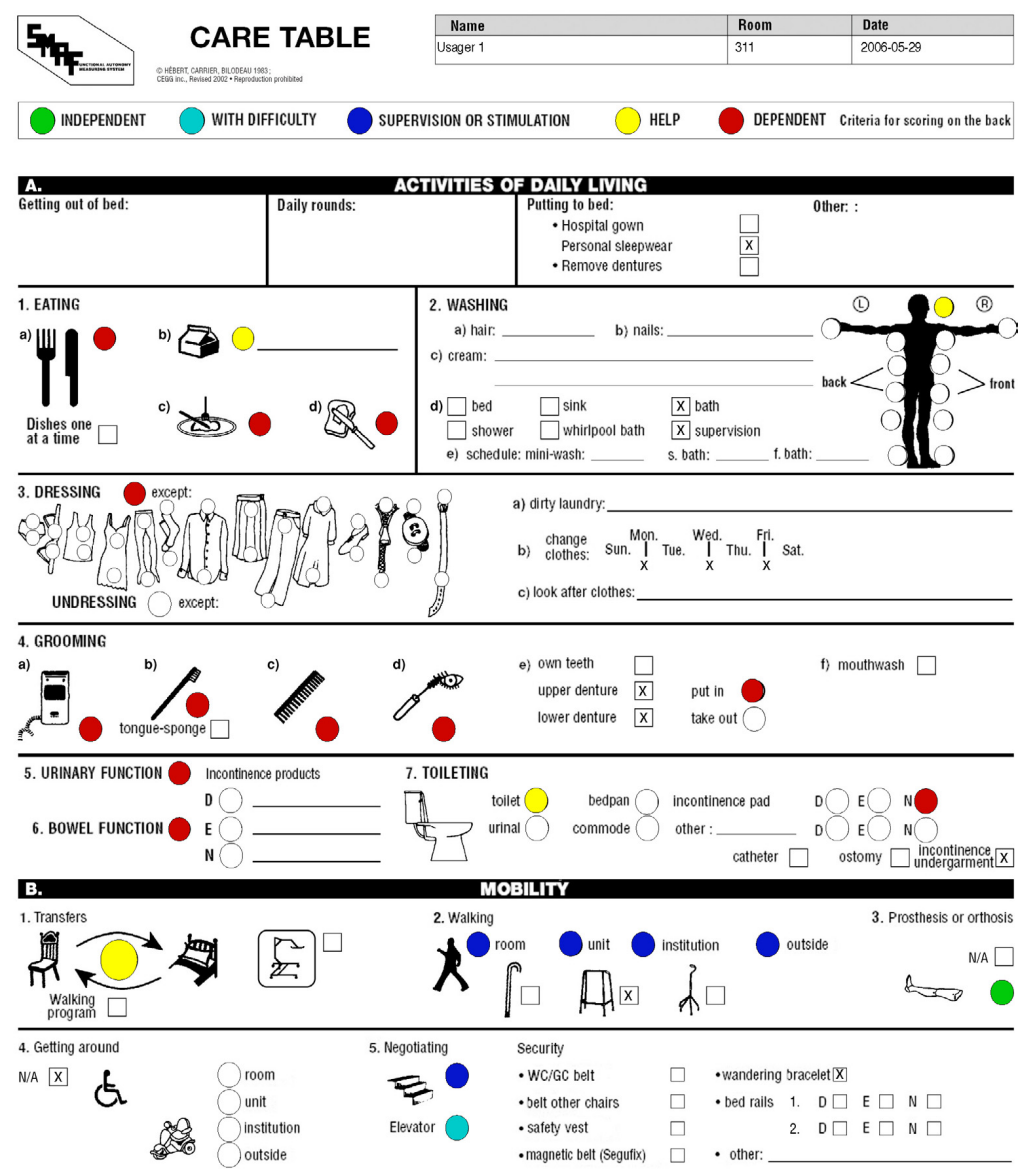
Care table generated from a SMAF evaluation.

In addition to these reporting features, users can automatically generate, for a given client or clients, a color-coded graphical care table that, for each section of the SMAF, summarizes the level of assistance needed by this or these individuals. The care table can be generated individually using data entered on the SMAF scale and printed. An illustration of a care table is presented in Figure 7. For a specific sphere of activities (ex: ADL), colors represent the level of assistance needed by the client for a given task (ex: bathing). The printed care table is then displayed in the client's room and serves as a visual reference to help guide the care provided to the client by nursing staff and orderlies.

#### Search functions

**Figure 8:**
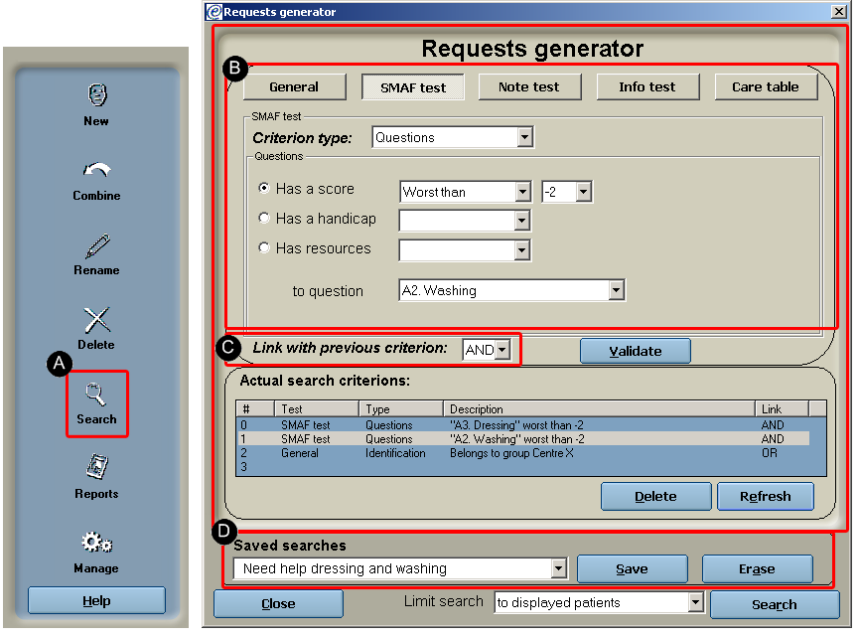
Search function. a) Activation of search function; b) Queries in the search function can be formulated on 5 categories of information; c) Queries can be linked together with logical operators, and d) saved as a specific search to be executed later.

Search functions were implemented at the aggregate client level to allow users to search and parse information from the database according to user-defined parameters. The search functions were implemented so that it would be possible to generate user-defined queries on all levels of the database (tests, patient information, groups, etc.) and execute report and data extraction functions on a subset of specific records. The interface for the search functions is illustrated in Figure 8. To do a search, search parameters need to be specified. There are no limits to the number of parameters that can be specified. Individual parameters are specified for a category of information (ex: score on a given section or question of the SMAF). Strings of parameters can be grouped together with the logical operator "And" or "Or" and saved as a specific search that can be reapplied at any time. Upon execution of a search, records (clients) matching the search parameters are clustered together as a group of records. Report functions and data extraction functions on this group of records or on individual records can then be applied.

#### Data extraction function and online help

**Figure 9:**
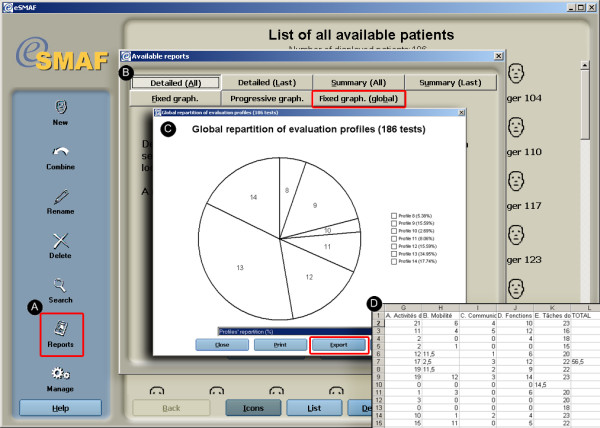
Data extraction function. a) Data extraction function; b) Data from the SMAF or c) specific section of the SMAF for a given individual or several individuals can be extracted to d) a CVS file.

**Figure 10:**
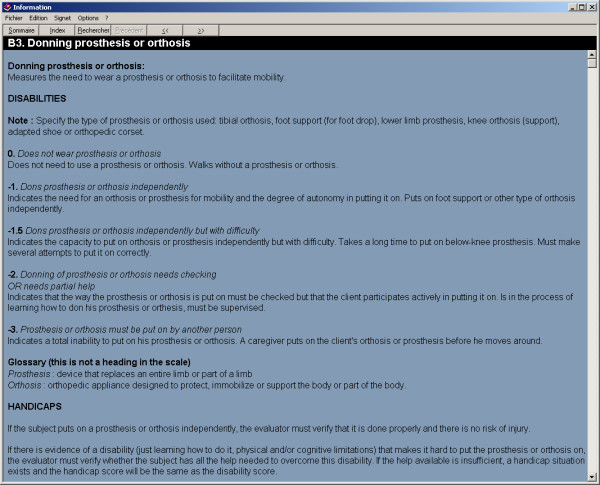
Online help function for scoring the SMAF.

Individual or aggregated group data can be transferred and merged with other datasets using the export function. Selected records or sections of records within the database can be exported as CVS files. The CVS file can be imported to another database, spreadsheet files and statistical applications such as SPSS. The data extraction function is illustrated in Figure 9. Online help in the eSMAF can be consulted to address issues with the software itself and to crosscheck scoring procedures on the SMAF evaluation. The software help system can be accessed from anywhere in the software. The SMAF help is only available when editing a SMAF test, and provides information on how to complete a specific question on the test. The help function is illustrated in Figure 10.

## Discussion

### Intended use of the software

The eSMAF's principal intended use is to streamline the collection of SMAF and Iso-SMAF data in clinical settings while offering a way to control the source and quality of the data entered, facilitate the aggregation, viewing and extraction of these data according to user-defined needs, and empower users to easily merge this information with other databases or data sources. For example, in the context of program evaluation research, the eSMAF was successfully deployed to collect SMAF outcome measures periodically over a 2-year period at 19 long-term public home care institutions in an administrative region of Quebec, Canada [24]. Medical archives personnel (n = 57 users) were trained on the use of the software prior to the start of the project. A key individual was identified in each facility to manage user questions and technical problems. This person was in contact with a district coordinator, who was the contact point for the research team. Clinical assessments in the eSMAF database from each institution were merged with administrative databases in order to objectively describe the relationship between the needs of older adults as established from Iso-SMAF profiles and the actual services provided to them by home care programs. SMAF data from 8000 patient records over a 2-year period were collected. As an example of the accessibility of these data, retrospective analysis of the data clearly illustrated the presence of a gap between the levels of home care services provided and the needs of disabled individuals, thus providing both researchers and clinical managers with arguments for changes in budget allocation for services in this administrative region. The data continue to be collected and used by administrative personnel to benchmark and document the autonomy of the client population and the services offered.

In this deployment, some barriers to implementation were encountered. The first was related to the use of small monitors and low resolution screens by some of the users. At the time many of the users were working with a screen resolution of 800 × 600 on 15-inch monitors. As the software interface was designed to work with a screen resolution of at least 1024 × 768, this caused a major problem. This problem was resolved by making the software automatically switch to the required resolution in the operating system (i.e. Windows) when launching, then switching back when it was closed. Another issue arose when the eSMAF was installed in many different network configurations in different physical locations, and regular updates were required because of its development stage. While the IT support needed to install and maintain the software in a server environment is minimal, this support was not readily available at most sites as IT support was contracted out. This introduced delays in granting user administrative and access privileges to the database. Communications with each site's IT support department was also difficult. When an update to the software was needed, it was not installed at the same time at each location. This caused problems when trying to find if a reported bug was related to a new or an old version and required close follow-up of installed versions in each physical location. Once installed and configured, the eSMAF performed as expected.

For clinical purposes, it is also possible to incorporate and analyze SMAF data and Iso-SMAF profiles on a regular basis, thereby rendering them accessible for decision-making in day-to-day operations (define eligibility criteria, support planning and evaluation processes, organize resource allocations) at different clinical and administrative levels. For example, from patient SMAF evaluations using the search function, department heads or hospital unit managers can estimate the clinical work load associated with a number of beds in a unit by looking at specific functional autonomy indicators (incontinence, dressing and eating) of patients in this unit. Care plans can also be circulated more freely and updated more easily. Prior to the development of the eSMAF, the clinical staff generated care tables for each client manually by putting color-coded stickers on each section of the care table and they had to crosscheck that they corresponded with the current SMAF data on record. The eSMAF's care table function offers an automated means to efficiently generate care tables for large numbers of clients while insuring that the outputs in these care tables correspond to the clients' on-record functional status.

### Planned future developments

Using feedback from current users, we are looking at optimizing the data management features of the eSMAF to facilitate its integration in institutional clinical workflow and/or its use in a health services research context. While the software in its current version has been used successfully in the context of multiple users (one database stored on a local area network with multiple controlled access to the database), the underlying database infrastructure is not optimized for large-scale networked use (i.e. database with more than 5000 records with simultaneous access from more than 40 users from multiple geographically separate sites). Initial user needs assessment and budgetary constraints governed the choice of the database infrastructure to a readily available non-commercial grade database format. The existing database platform (Microsoft Access 2000) is not robust enough to operate under such conditions. Therefore, to expand the usability of the software under this context of use, a Web-based eSMAF supported with a commercial grade database format (ex: My SQL) is being considered.

Dynamic integration of the eSMAF software with existing clinical information systems will also a play a critical role in its successful deployment in large-scale hospital settings. This integration is possible with the existing software architecture but depends to a large extent on establishing communication between systems and establishing database synchronization rules that fit the workflow and needs of the application. Depending on user interest, another development area will be to offer other language versions. As the SMAF instrument has been translated into 7 languages, the eSMAF could be ported to other languages. The underlying programming architecture related to the operation and use of the software is not language-specific. Indeed the programming architecture is such that most of the user interface shell (data entry mask of the SMAF) can be customized rapidly while still maintaining the current database structure using tables containing content in any language.

## Conclusion

The eSMAF software was developed with limited resources to address specific needs. It can be used in different research and clinical contexts to optimize and facilitate the assessment and follow-up of elderly disabled patients with the Functional Autonomy Measurement System and Iso-SMAF profiles. The robustness and usability of the software were tested through iterative field trials with users. An academic license for the software is now available to qualified users. Maintenance of the existing version of the software will be executed on a limited basis as permitted by the availability of new financial resources through institutional licensing.

## Availability and requirements

Project name: eSMAF

Operating system(s): Microsoft 2000, XP

Programming language: Visual Basic

Other requirements: Monitor resolution of 1024 × 768

Licenses: Full version 30-day demonstration, restricted academic license through e-mail activation of demonstration version. Commercial license needed for non-academic or institutional use.

Demonstration license: Time-limited demonstration version through direct download from BMC geriatrics.  English (Additional file 3; French (Additional file 4)

Registered academic license: Restricted free academic licenses can be obtained by contacting the Sherbrooke Health Expertise Center at info@expertise-sante.com.



Commercial license: For institutional and/or commercial use, licensing inquiries can also be made to the Sherbrooke Health Expertise Center at info@expertise-sante.com.



## Competing interests

The eSMAF is the property of the Sherbrooke Health Expertise Center. Patrick Boissy, PhD, was funded by the Sherbrooke Health Expertise Center to develop and test the software. Simon Brière received a salary as an intern for an 12-month period from this funding during the development of the software.

## Authors' contributions

PB participated in the design, development and evaluation of the software, and drafted the manuscript. SB participated in the development of the software and drafted the manuscript. MT contributed to the coordination of the field evaluation of the software and reviewed the manuscript. ER participated in the design and development of the software and reviewed the manuscript. All the authors read and approved the final manuscript.

**Appendix 1**. a) Areas of functional ability evaluated by the SMAF; b) Illustration of scoring template for an item on the SMAF.

**Appendix 2**. Summary of ISO-SMAF case-mix classification profiles according to patients' functional autonomy characteristics on the SMAF.

## Pre-publication history

The pre-publication history for this paper can be accessed here:



## Supplementary Material

Additional File 1 Appendix 1. Areas of functional ability evaluated by the SMAF and scoring template for an item. For more details on the SMAF, see Hebert R, Carrier R, Bilodeau A: The Functional Autonomy Measurement System (SMAF): description and validation of an instrument for the measurement of handicaps. *Age Ageing *1988, 17(5):293–302. Click here for file

Additional File 2 Appendix 1. Summary of Iso-SMAF case-mix classification profiles according to patients' functional autonomy characteristics on the SMAF. For more details on the Iso-SMAF case mix classification system, see Dubuc N, Hebert R, Desrosiers J, Buteau M: Long-term care for the elderly: choice of a clinical managerial system in the context of an integral care network. *Can J Aging *2004, 23(1):35–45. Click here for file

Additional File 3 eSMAF download (English). Click here for file

Additional File 4 eSMAF download (French). Click here for file

## References

[B1] Shortell S, Gillies R, Anderson D, Erickson K, Mitchell J (2000). Remaking health care in America: The evolution of organized delivery systems.

[B2] Hébert R, Carrier R, Bilodeau A (1988). The Functional Autonomy Measurement System (SMAF): description and validation of an instrument for the measurement of handicaps. Age Ageing.

[B3] Hébert R, Guilbault J, Desrosiers J, Dubuc N (2001). The Functional Autonomy Measurement System (SMAF): A clinical-based instrument for measuring disabilities and handicaps in older people. Journal of the Canadian Geriatrics Society.

[B4] Direction des communications du ministère de la Santé et des Services sociaux (2000). Recommandations du Comité aviseur sur l'outil d'évaluation intégré des besoins des personnes en perte d'autonomie et de détermination des services requis. Quebec.

[B5] World Health Organization (1980). International classification of impairments, disabilities and handicaps: a manual of classification relating to the consequences of disease. Geneva.

[B6] Hébert R (1997). Functional decline in old age. CMAJ.

[B7] Grill E, Hermes R, Swoboda W, Uzarewicz C, Kostanjsek N, Stucki G (2005). ICF Core Set for geriatric patients in early post-acute rehabilitation facilities. Disabil Rehabil.

[B8] Ustun TB, Chatterji S, Bickenbach J, Kostanjsek N, Schneider M (2003). The International Classification of Functioning, Disability and Health: a new tool for understanding disability and health. Disabil Rehabil.

[B9] Pinsonnault E, Desrosiers J, Dubuc N, Kalfat H, Colvez A, Delli-Colli N (2003). Functional autonomy measurement system: development of a social subscale. Arch Gerontol Geriatr.

[B10] Rai GS, Gluck T, Wientjes HJ, Rai SG (1996). The Functional Autonomy Measurement System (SMAF): a measure of functional change with rehabilitation. Arch Gerontol Geriatr.

[B11] Langlais M (1997). Estimation et comparaison de la sensibilité au changement de trois échelles d'indépendance fonctionnelle couramment utilisées en réadaptation gériatrique. Thesis.

[B12] Hébert R, Spiegelhalter DJ, Brayne C (1997). Setting the minimal metrically detectable change on disability rating scales. Arch Phys Med Rehabil.

[B13] Desrosiers J, Bravo G, Hebert R, Dubuc N (1995). Reliability of the revised functional autonomy measurement system (SMAF) for epidemiological research. Age Ageing.

[B14] Hébert R, Bilodeau A (1986). Profil d'autonomie des personnes âgées hébergées en institution. Cah Assoc Can Fr Av Sci.

[B15] Dubuc N, Hébert R, Desrosiers J, Buteau M (2004). Long-term care for the elderly: choice of a clinical managerial system in the context of an integral care network. Can J Aging.

[B16] Tousignant M, Hebert R, Dubuc N, Simoneau F, Dieleman L (2003). Application of a case-mix classification based on the functional autonomy of the residents for funding long-term care facilities. Age Ageing.

[B17] Dubuc N, Hebert R, Desrosiers J, Buteau M, Trottier L (2006). Disability-based classification system for older people in integrated long-term care services: the Iso-SMAF profiles. Arch Gerontol Geriatr.

[B18] Sambrook R, Herrmann N, Hébert R, McCracken P, Robillard A, Luong D, Yu A (2004). Canadian Outcomes Study in Dementia: study methods and patient characteristics. Can J Psychiatry.

[B19] Hébert R, Robichaud L, Roy PM, Bravo G, Voyer L (2001). Efficacy of a nurse-led multidimensional preventive programme for older people at risk of functional decline. A randomized controlled trial. Age Ageing.

[B20] Lefrancois R, Hébert R, Dube M, Leclerc G, Hamel S, Gaulin P (2000). Incidence of the onset of disability and recovery of functional autonomy among the very old after one year. Rev Epidemiol Sante Publique.

[B21] Bravo G, Charpentier M, Dubois MF, DeWals P, Emond A (1998). Profile of residents in unlicensed homes for the aged in the eastern townships of Quebec. CMAJ.

[B22] Waterfall model. http://en.wikipedia.org/wiki/Waterfall_model.

[B23] Abran A, Khelifi A, Suryn W, Seffah A (2003). Usability Meanings and Interpretations in ISO Standards. Software Quality Journal.

[B24] Tousignant M, Dubuc N, Hébert R, Coulombe C (2007). Home-care programmes for older adults with disabilities in Canada: How can we assess the adequacy of services provided compared with the needs of users?. Health Soc Care Community.

