# Supplementary material for: The eSMAF: a software for the assessment and follow-up of functional autonomy in geriatrics
Source: BMC Geriatr. 2007 Feb 13;7:2. doi: 10.1186/1471-2318-7-2 (PMC1802868; doi:10.1186/1471-2318-7-2)
Supplement: Additional File 1 — Appendix 1. Areas of functional ability evaluated by the SMAF and scoring template for an item. For more details on the SMAF, see Hebert R, Carrier R, Bilodeau A: The Functional Autonomy Measurement System (SMAF): description and validation of an instrument for the measurement of handicaps. Age Ageing 1988, 17(5):293–302. [file 1471-2318-7-2-S1.pdf]

A

A. Activity of daily living (ADL)	D. Mental functions
1. Eating	1. Memory
2. Bathing	2. Orientation
3. Dressing	3. Understanding
4. Grooming	4. Judgement
5. Urinary continence	5. Behavior
6. Faecal continence	
7. Using the bathroom	
**B. Mobility**	**E. Instrumental activities of daily living**
1. Transferts	1. Cleaning the house
2. Walking inside	2. Preparing meals
3. Walking outside	3. Shopping
4. Putting on prothesis or orthosis	4. Doing the laundry
5. Moving around in a wheelchair	5. Using the phone
6. Using the stairs	6. Using public transportation
	7. Taking medication
**C. Communication**	8. Managing the budget
1. Seeing	
2. Hearing	
3. Talking	

B

DISABILITIES	RESOURCES	HANDICAP	STABILITY*
	0. Subject himself<br>1. Family<br>2. Neighbour<br>3. Employee<br>4. Aides<br>5. Nurse<br>6. Volunteer<br>7. Other		

A. ACTIVITIES OF DAILY LIVING (ADL)

1. EATING

- [0] Feeds self independently
  - [-0,5] With difficulty
- [-1] Feeds self but needs stimulation or supervision OR food must be prepared or cut or pureed first
- [-2] Needs some help to eat OR dishes must be presented one after another
- [-3] Must be fed totally by another person OR has a naso-gastric tube or a gastrostomy
  - ☐ naso-gastric tube ☐ gastrostomy

Does the subject presently have the human resources (help or supervision) necessary to overcome this disability?

- ☐ Yes → [0]
- ☐ No → [-1] [-2] [-3]

Resources: ☐ ☐ ☐

Stability: [−] [+] [•]
