# Supplementary material for: The eSMAF: a software for the assessment and follow-up of functional autonomy in geriatrics
Source: BMC Geriatr. 2007 Feb 13;7:2. doi: 10.1186/1471-2318-7-2 (PMC1802868; doi:10.1186/1471-2318-7-2)
Supplement: Additional File 2 — Appendix 1. Summary of Iso-SMAF case-mix classification profiles according to patients' functional autonomy characteristics on the SMAF. For more details on the Iso-SMAF case mix classification system, see Dubuc N, Hebert R, Desrosiers J, Buteau M: Long-term care for the elderly: choice of a clinical managerial system in the context of an integral care network. Can J Aging 2004, 23(1):35–45. [file 1471-2318-7-2-S2.pdf]

Appendix 2

Profil #1	Profil #8
Need supervision for housekeeping and occasional help for heavy housework; difficulty with meal preparation, using transportation and managing budgeting.	Moderate alterations in cognitive functions; unable to make decisions; minor behavioural problems; difficulty in ADL; need help for washing and grooming; need supervision in mobility functions.
**Profil #2**	**Profil #9**
Need help to do laundry, daily housework and preparing meals, shops but need delivery service, difficulty using transportation and managing budgets.	Severe alterations in mobility functions; may need help in washing, dressing and grooming; may need a wheelchair; present occasional incontinence; very minor deficits in all mental functions.
**Profil #3**	**Profil #10**
Completely dependant for housekeeping, preparing meals, shopping and laundry; can answer telephone only with memorized or emergency numbers. Minor recent memory deficit. Difficulties in washing and grooming.	Severe alterations in cognitive functions with moderate behavioral problems; need help in ADL, but walk independently inside; need supervision for toilet; problems expressing themselves (speech/language problems).
**Profil #4**	**Profil #11**
Minor alterations in mobility functions; need regular IADL help; mainly independent in ADL, except for washing and grooming; independent in mental functions.	Mixed and severe alterations in mobility and cognitive functions; need help for transfers; dependent in washing, dressing and grooming; maintain bowel functions, but need help using the toilet; most in wheelchairs.
**Profil # 5**	**Profil #12**
Minor alterations in some cognitive functions, but poor judgment; dependent in IADL; difficulty washing and grooming; independent in mobility.	Mixed and severe alterations in mobility and cognitive functions; major behavioral problems; need help for washing, dressing and grooming; total incontinence; need help in mobility; minor sensory deficits.
**Profil #6**	**Profil #13**
Moderate alterations in mobility functions; difficulty eating and dressing; need help with washing and grooming; mainly independent mental functions, but minor deficits in memory and judgment.	13. Mixed and very severe alterations in mobility and cognitive functions; bedridden and dependency in ADL; severe cognitive impairment, but minor behavioral problems.
**Profil #7**	**Profil #14**
Moderate alterations in cognitive functions; unable to make decisions; minor behavioral problems; difficulty in ADL; need help for washing and grooming. Need supervision to walk outside.	Mixed and very severe alterations in mobility and cognitive functions; bedridden and dependency in ADL; severe cognitive impairment; moderate behavioural problems; moderate sensory deficits; unable to communicate.
